# SARS-CoV-2 as a potential trigger for microscopic colitis in a young adult: A case report

**DOI:** 10.1016/j.heliyon.2024.e35086

**Published:** 2024-07-23

**Authors:** A. Alwali, C. Schafmayer, F. Prall, I. Kamaleddine

**Affiliations:** a Department of General, Visceral, Thorax, Vascular and Transplantation Surgery, Rostock University Medical Center, Rostock, Germany; b Institute of Pathology, Rostock University Medical Center, Rostock, Germany

**Keywords:** Lymphocytic colitis, Chronic diarrhea, Microscopic colitis, SARS-CoV-2, Case report

## Abstract

This case highlights the potential role of SARS-CoV-2 in triggering lymphocytic colitis, emphasizing the need for further research and vigilance in identifying potential post-COVID-19 GI complications. We describe a case of a young adult who experienced chronic diarrhea and abdominal pain for 10 months after a SARS-CoV-2 infection. Extensive laboratory and imaging investigations yielded no significant findings. Despite a preliminary diagnosis of irritable bowel syndrome and symptomatic treatment, symptoms persisted. Colonoscopy with biopsies revealed unremarkable colonic mucosa but confirmed moderate lymphocytic infiltration consistent with lymphocytic colitis. Treatment with budesonide achieved complete symptom resolution. The findings underscore the importance for clinicians to consider triggered microscopic colitis in patients presenting with persistent diarrhea following SARS-CoV-2 infection.

## 1 Introduction

Microscopic colitis (MC), a chronic inflammatory condition affecting the colon, is experiencing a rising incidence. Based on biopsy findings, there are two types of microscopic colitis: collagenous and lymphocytic colitis. The incidence of microscopic colitis has increased over time ranges from of 4.9 (95 % CI 4.2–5.7) cases per 100,000 patient-years for collagenous colitis and 5.0 (95 % CI 4.0–6.1) cases per 100,000 patient-years for lymphocytic colitis, with higher prevalence in northern Europe and the northern regions of North America [1]. MC primarily affects older adults around the age of 65, although approximately 25 percent of cases are identified before the age of 45 [2]. While rare in children, there is a female predilection in both collagenous and lymphocytic colitis. The hallmark feature is chronic or intermittent watery non-bloody diarrhea, accompanied by urgency, abdominal discomfort and normal or almost normal mucosal appearance on colonoscopy. There is no particular biomarker available for diagnosing microscopic colitis. Therefore, confirmation currently relies on examining tissue samples obtained through colonoscopy. While infectious agents causing episodes of infectious gastroenteritis may play a role in initiating and/or exacerbating inflammatory bowel disease (IBD), there is limited data linking SARS-CoV-2 infection as a trigger for microscopic colitis [3].

## 2 Case presentation

**Fig. 1:**
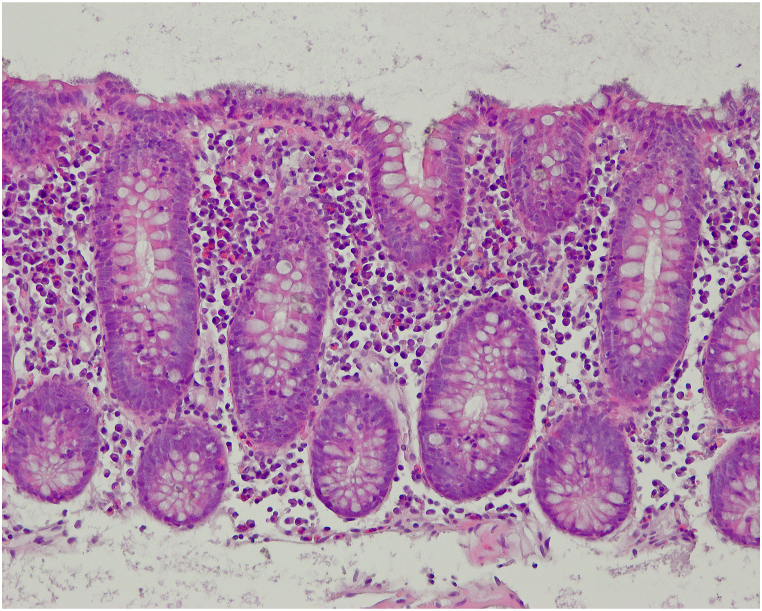
Representative microscopic image of the colonic mucosa. Note the intraepithelial lymphocytosis which was seen in all biopsies.

**Fig. 2:**
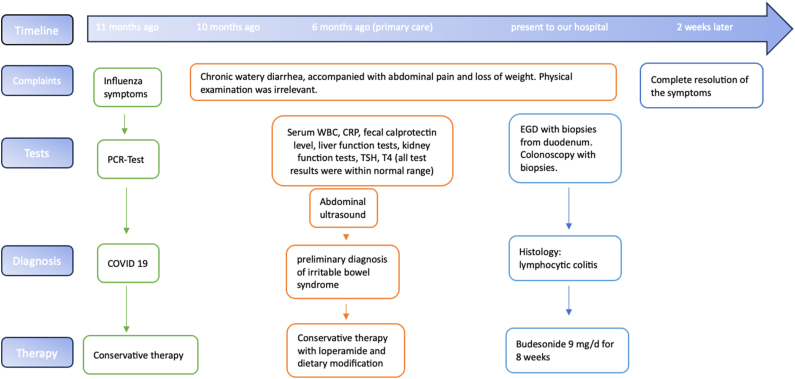
Timeline of the patient's complaints, clinical findings, tests results, diagnosis and interventions. Abbreviations: PCR, Polymerase chain reaction test; COVID 19, Coronavirus disease 2019; WBC, white blood cell; EGD, Esophagogastroduodenoscopy.

A 39-year-old male presented to our department for evaluation and treatment of chronic loose diarrhea, accompanied with recurrent abdominal pain lasting for 10 months. Additionally, he reported a weight loss of 6 kg during this period. His medical history revealed a past SARS-CoV-2 infection 6–8 weeks prior to his current symptoms. However, he did not directly attribute his symptoms to this prior SARS-CoV-2 infection. Furthermore, the patient had received his last dose of the SARS-CoV-2 mRNA vaccine approximately 18 months before his presentation to us. Aside from these occurrences, his medical history and examination revealed no other significant findings. Notably, there were no reports of fever, blood or pus in the stools, medication intake, or family history of inflammatory bowel disease (IBD). The symptoms appeared more pronounced during periods of stress at work. The initial laboratory tests, including blood counts, C-reactive protein, fecal calprotectin level, liver function tests, kidney function tests, stool microscopy and cultures, and thyroid function tests, provided no significant findings. Abdominal ultrasound also showed no abnormalities. Six months prior, the patient sought medical advice from primary care, resulting in a preliminary diagnosis of irritable bowel syndrome. Symptomatic treatment with loperamide and a recommended dietary modification showed no success. We decided to perform an esophagogastroduodenoscopy and colonoscopy to complete the evaluation. Esophagogastroduodenoscopy and biopsies from the stomach and duodenum revealed no abnormalities, providing no evidence for coeliac disease or Whipple's disease. Colonoscopy showed an unremarkable mucosa in the colon and terminal ileum. Mucosal biopsies were taken from the entire colon to rule out microscopic colitis. Histology revealed a moderate but definitely abnormal lymphocytic infiltration of the colonic surface epithelium throughout the entire colonic mucosa (Fig. 1) which otherwise was essentially unremarkable. Importantly, there was not observed any increase of subepithelial collagen. Based on the combination of the clinical presentation of chronic watery diarrhea, normal colonoscopic examination, and typical microscopic findings, a diagnosis of lymphocytic colitis was established. The patient was prescribed an 8-week course of oral budesonide 9 mg once a day for induction of remission. Complete resolution of symptoms was noted after few days, and clinical remission has been maintained so far. The treatment was well tolerated, with no reported adverse or unexpected events. The timeline of the patient's complaints, clinical findings, tests results, diagnosis and interventions applied are summarized in Fig. 2.

## 3 Discussion

Microscopic colitis symptom severity varies widely, with some individuals experiencing mild, occasional episodes and others struggling with persistent, debilitating symptoms. In severe cases, electrolyte imbalances and dehydration can pose additional health risks. The disease course itself is equally unpredictable, ranging from spontaneous remission to chronic, recurrent manifestations. While some patients find relief without intervention, others require ongoing maintenance therapy to manage their symptoms effectively [4]. Due to the considerable symptom load and significant impact on quality of life, it's crucial to accurately distinguish between patients with microscopic colitis and those with irritable bowel syndrome.

Several factors increase the risk of developing MC. These include advanced age, female sex, smoking and the presence of other autoimmune diseases. Certain medications, such as proton pump inhibitors (PPIs), nonsteroidal anti-inflammatory drugs (NSAIDs) and selective serotonin reuptake inhibitors (SSRIs), may also play a role [5]. The underlying mechanisms of microscopic colitis remain poorly understood, yet it is probable a result of a complex interplay of factors. These include immune system imbalances, epithelial dysfunction, collagen metabolism abnormalities, secretory diarrhea, and gut microbiota alterations, combined with the risk factors mentioned above in genetically predisposed individuals [[6], [7], [8], [9]]. While others have extensively explored this intricate domain, our focus in this case report is directed towards recent insights within the framework of SARS-CoV-2. Infectious agents are believed to induce autoimmunity through mechanisms such as molecular mimicry, bystander activation, polyclonal activation, and epitope spreading [10]. Among these, molecular mimicry is considered the most likely mechanism. This occurs when there is cross-reactivity between epitopes—proteins, carbohydrates, or DNA—shared by the pathogen and the host, leading the immune system to mistakenly attack the body's own tissues [10]. It is speculated that SARS-CoV-2 can disrupt self-tolerance and trigger autoimmune responses through cross-reactivity with host cells, and that the COVID-19 mRNA vaccines might induce a similar response [11]. Reports suggest that the SARS-CoV-2 spike protein can interact with different human tissue antigens [12]. It is also speculated that adverse events following vaccination could be linked to the spike protein present in both the virus and the vaccines [13].

Additionally, other vaccines have been associated with the development of autoimmune diseases, such as narcolepsy, Guillain-Barré syndrome, multiple sclerosis, demyelinating neuropathy, systemic lupus erythematosus, and postural orthostatic tachycardia syndrome [14].

Chey et al. reported a case of lymphocytic colitis after mRNA vaccination and mentioned a Review of the Vaccine Adverse Event Reporting System identifying five cases of mRNA vaccine-associated microscopic colitis following both Pfizer-BioNTech and Moderna vaccinations. Among these cases, three individuals experienced severe diarrhea shortly after their second mRNA vaccine dose, while two others developed diarrhea approximately one month after their second dose [15]. Lee et al. reported a case of lymphocytic colitis that occurred in a healthy middle-aged man after Moderna SARS-CoV-2 mRNA vaccination [16]. Nassar et al. and Moiteiro da Cruz et al. each reported cases of lymphocytic colitis and collagenous colitis, respectively, following SARS-CoV-2 infection [17,18]. In the absence of other identifiable risk factors for microscopic colitis, we hypothesize that the prior SARS-CoV-2 infection in our case may have triggered his current condition by activating the innate immune system.

## 4 Clinical relevance

This case highlights the significance for healthcare providers to include microscopic colitis in their differential diagnosis when evaluating patients with persistent diarrhea, particularly in the context of recent SARS-CoV-2 infection or vaccination. However, further research is warranted to better understand this association.

## Data availability statement

No data was used for the research described in the article.

## Additional information

No additional information is available for this paper.

## Informed consent

The authors certify that they have obtained all appropriate patient consent forms. In the form, the patient has given consent for his histological image and other clinical information to be reported in the journal.

## Fundings

This research did not receive any funding from agencies in the public, commercial, or not-for-profit sectors.

## CRediT authorship contribution statement

**A. Alwali:** Writing – review & editing, Writing – original draft, Methodology, Data curation, Conceptualization. **C. Schafmayer:** Validation, Supervision, Data curation. **F. Prall:** Writing – review & editing, Investigation, Data curation. **I. Kamaleddine:** Writing – review & editing, Data curation.

## Declaration of competing interest

The authors declare that they have no known competing financial interests or personal relationships that could have appeared to influence the work reported in this paper.
